# Functionalization and Evaluation of Inorganic Adsorbents for the Removal of Cadmium in Wastewater

**DOI:** 10.3390/molecules26144150

**Published:** 2021-07-08

**Authors:** Ana Lucía Campaña, Amaimen Guillén, Ricardo Rivas, Veronica Akle, Juan C. Cruz, Johann F. Osma

**Affiliations:** 1 Department of Electrical and Electronic Engineering, Universidad de Los Andes, Bogotá 111711, Colombia; al.campana10@uniandes.edu.co (A.L.C.); aa.guillon@uniandes.edu.co (A.G.); 2 Laboratory of Neuroscience and Circadian Rhythms, School of Medicine, Universidad de Los Andes, Bogotá 111711, Colombia; v.akle@uniandes.edu.co; 3 Department of Chemistry, Science Faculty, Universidad de Los Andes, Bogotá 111711, Colombia; re.rivas@uniandes.edu.co; 4 Department of Biomedical Engineering, Universidad de Los Andes, Bogotá 111711, Colombia; jc.cruz@uniandes.edu.co

**Keywords:** wastewater, cadmium, magnetite nanoparticles, alumina, functionalization

## Abstract

This study presents the feasibility of using various functionalized substrates, Fe_3_O_4_ nanoparticles (NPs) and Al_2_O_3_ spheres, for the removal of Cd from aqueous solution. To improve the materials’ affinity to Cd, we explored four different surface modifications, namely (3-Aminopropyl) triethoxysilane (APTES), L-Cysteine (Cys) and 3-(triethoxysilyl) propylsuccinic anhydride (CAS). Particles were characterized by FTIR, FIB-SEM and DLS and studied for their ability to remove metal ions. Modified NPs with APTES proved to be effective for Cd removal with efficiencies of up to 94%, and retention ratios up to 0.49 mg of Cd per g of NPs. Batch adsorption experiments investigated the influence of pH, contact time, and adsorbent dose on Cd adsorption. Additionally, the recyclability of the adsorbent and its potential phytotoxicity and animal toxicity effects were explored. The Langmuir, Freundlich, pseudo-first-order and pseudo-second-order models were applied to describe the behavior of the Cd adsorption processes. The adsorption and desorption results showed that Fe_3_O_4_ NPs modified with APTES are promising low-cost platforms with low phytotoxicity for highly efficient heavy metal removal in wastewater.

## 1. Introduction

Cadmium (Cd) is a relatively scarce heavy metal found in the earth’s crust and the inevitable product of the extraction of zinc, lead, and copper. In the environment, Cd only exists in one oxidation state (+2), dissolving easily in polluted waters with low pH levels and typically transported to freshwaters used for human consumption [1,2]. One of the main sources of Cd contamination is poorly treated domestic, industrial and agricultural wastewater which usually contains high concentrations of heavy metals [1]. The concentrations of this element in wastewater have been reported to vary from 0 to 1000 mg/L, depending on the discharge source [3]. This is a major environmental and health concern because the established maximum level of this pollutant according to the U.S. Environmental Protection Agency (US EPA) is 0.005 mg/L for drinking water [4] and, according to the International Agency for Research on Cancer (IARC), Cd is linked to kidney damage, chronic renal failure, osteoporosis, and cancer, mainly due to its marked tendency for bioaccumulation [1,5,6]. Thus, Cd traces must be effectively removed before the discharge of wastewater into the environment [7,8].

Several Cd removal methods have been studied and implemented on an industrial scale, including chemical precipitation [3], solvent extraction [9,10], flocculation [11], electrochemical treatment [12], membrane filtration [13] and adsorption. Among them, adsorption-based processes are considered simple, cost-effective, and sustainable alternatives for heavy metal removal. For this, some of the preferred adsorbents include different variations of activated carbon [14], carbon nanotubes [15,16], various nanoparticles [17,18,19,20] and agricultural waste by-products such as rice husk, fly ash [21], sunflower waste [22,23] and chitosan [24,25]. The attractiveness of these materials as adsorbents stems from the fact that they exhibit a high surface area, high adsorption capacity, mechanical stability, and ease of recovery. Despite their potential, many of these substrates show insufficient superficial active sites and their specificity towards the effective removal of metal ions is highly limited. To overcome this issue, recent nanotechnology approaches have provided novel surface modification routes to introduce functional groups that show a higher affinity for Cd chelation and consequently, are capable of considerably increasing the sorption capacity of these materials [26,27,28]. The Hosain et al. study on magnetic nanoparticles cross-linked succinic anhydride reported a maximum removal efficiency of Cd from wastewater samples of 57.6% [29] and Chen et al. [30] a removal of 96% using functionalized Fe_3_O_4_ nanoparticles. For nanomaterials based on alumina (Al_2_O_3_), the maximum removal efficiencies reported are between 31% and 99% [31,32]. These, however, rarely include the recovery studies and toxicity analysis to determine the viability of using the related components on a larger scale.

Despite the ample number of emerging treatments, much work is still needed to ensure effective water treatment processes that are also economically feasible and sustainable in the long run. Hence, the purpose of this work is to evaluate the efficiency of nanocomposite materials as practical treatment options. In this work, two inorganic substrates, Al_2_O_3_ spheres and Fe_3_O_4_ nanoparticles (NPs) with four different surfaces modifications are proposed for Cd adsorption. Here, we evaluated the effect of pH, contact time, and substrate dose on Cd removal. Additionally, one of the adsorbents was analyzed for recyclability, as well as for the environmental repercussions of their use.

## 2. Results

The study of the removal of Cd from water was carried out using two distinct inorganic surfaces, commercial Al_2_O_3_ spheres of 600–800 µm diameter and produced Fe_3_O_4_ NPs. These substrates were selected as they both can be easily retrieved from liquid environments which makes them ideal materials for large scale heavy metal treatment processes. Additionally, different surface functionalizations were prepared to evaluate their effect on the Cd removal efficiency. The substrates were functionalized by silanization with two different organo-silane molecules, APTES and CAS, then further functionalized with L-Cysteine (Cys). The final evaluated substrates were the original materials and the adsorbents modified with CAS, CAS@Cys, APTES, and APTES@Cys. The adsorbent with the best recovery and removal results was then further evaluated at different pH values, contact time and adsorbent dose. Two kinetic and isotherm models of this adsorbent were also fitted to the obtained data.

### 2.1. Characteristics of the Adsorbents

#### 2.1.1. Dynamic Light Scattering (DLS) of Fe_3_O_4_ NPs

**Figure 1:**
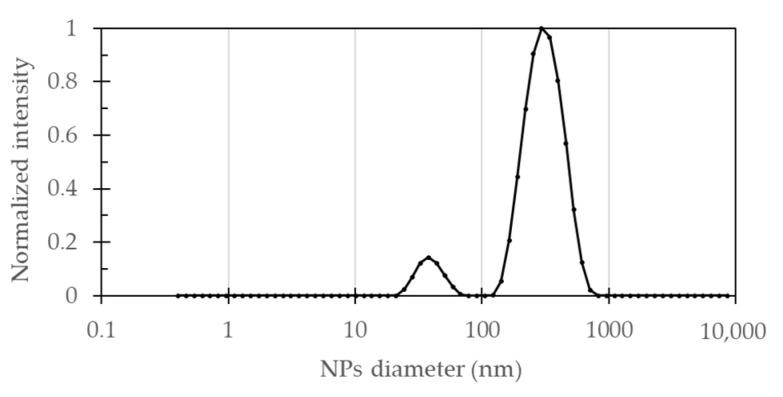
DLS result of particle size distribution for bare Fe_3_O_4_ NPs nanoparticles in aqueous suspension. The first peak from left to right is centered at 39.4 ± 9.1 nm (8.9%) while the second one is at 323.6 ± 107.2 nm (91.1%).

The size distribution of the synthesized Fe_3_O_4_ NPs was measured using DLS. Figure 1 shows that the particle size distribution of bare Fe_3_O_4_ NPs solution presented two main peaks centered at 39.4 ± 9.1 nm (8.9%) and 323.6 ± 107.2 nm (91.1%). A polydispersity index (PDI) of 0.329 indicated that the synthesized particles exhibited uniform sizes. Most of the NPs exhibited a size somewhat larger than expected for this synthesis method, which was attributed to particle agglomeration processes. This can also be inferred from the standard deviation of largest peak that was significantly wider than expected for a monodisperse sample.

#### 2.1.2. Focused Ion Beam-Scanning Electron Microscopy (FIB-SEM) with EDX Analysis

**Figure 2:**
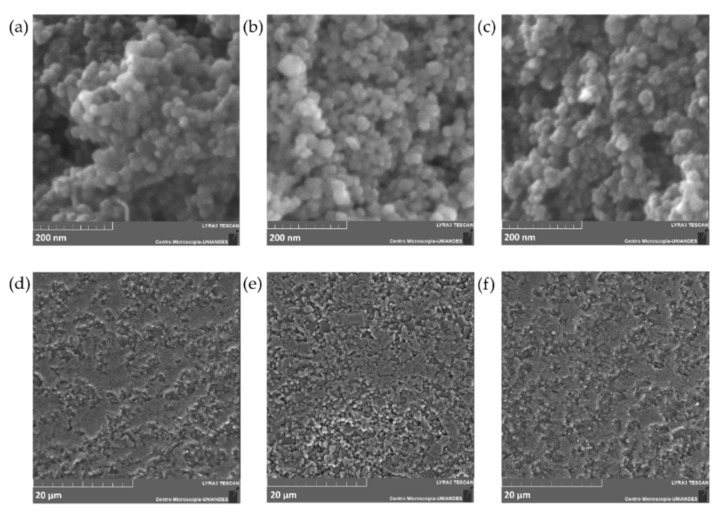
SEM images of (**a**) Fe_3_O_4_ NPs, (**b**) Fe_3_O_4_ NPs @APTES, (**c**) Fe_3_O_4_ NPs after Cd removal, (**d**) Al_2_O_3_ spheres, (**e**) Al_2_O_3_ @APTES, and (**f**) Al_2_O_3_ spheres after Cd removal.

The morphology and size of the adsorbents before and after Cd removal experiments were studied via FIB-SEM. Figure 2 shows the corresponding micrographs for the adsorbents prior and after modification with (3-Aminopropyl) triethoxysilane (APTES), and after Cd removal. The micrographs for the bare Fe_3_O_4_ NPs (Figure 2a,b) show a typical spherical and monodispersed morphology, however, unfunctionalized particles appear to have higher agglomeration in contrast with the APTES-modified NPs (Figure 2a–c). The mean size of bare Fe_3_O_4_ NPs is approximately 10–30 nm, which agrees with one of the peaks found with the DLS analysis. No apparent change in the NPs diameter was observed after Cd removal experiments.

Figure 2d–f show a rough surface for the 600–800 µm Al_2_O_3_ spheres, which is usually observed for high superficial area and superficially porous materials [33,34]. However, a closer inspection of the surface revealed a highly dense material with low porosity, which most likely indicates that the spheres have a much lower surface-active area for functionalization than the NPs counterparts.

**Figure 3:**
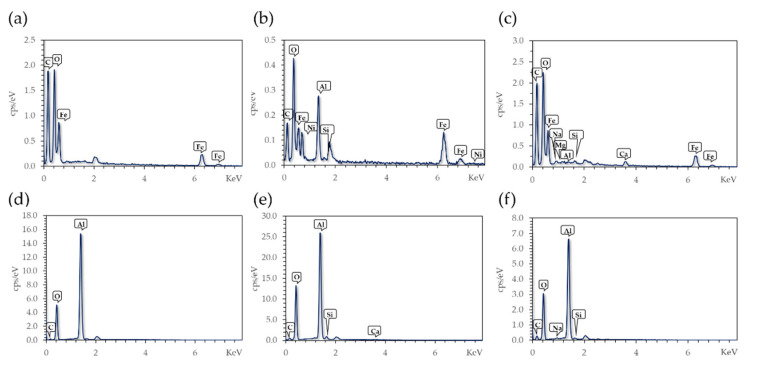
EDX results of (**a**) Fe_3_O_4_ NPs, (**b**) Fe_3_O_4_ NPs @APTES, (**c**) Fe_3_O_4_ NPs @APTES after Cd removal, (**d**) Al_2_O_3_ spheres, (**e**) Al_2_O_3_ spheres @APTES, and (**f**) Al_2_O_3_ spheres @APTES after Cd removal.

The EDX results shown in Figure 3 confirmed the modification with the organo-silanes, APTES and 3-(triethoxysilyl) propylsuccinic anhydride (CAS) on Fe_3_O_4_ NPs and Al_2_O_3_ spheres. The presence of silicon (Si) in the spectrum after modification strongly indicates a correct functionalization with the organosilane molecules; however, in the case of Al_2_O_3_ spheres, the presence of Si is frequently related to initial traces of silicon on the material. However, in the control sample there was no evidence of a Si peak, which is why the Si in the modified material can be related to surface functionalization, not the original sample composition. 

Samples after Cd treatment were also evaluated via EDX (Figure 3c,f). There were no traces of Cd in any of the studied substrates. The initial concentration of Cd for the adsorption experiments was 0.1 mg/L, and consequently, the adsorbed amount by the materials was expected to be lower than 1% on weight basis. This poses a difficulty in the detection of adsorbed Cd, as the resolution of this method is inadequate for this limit.

#### 2.1.3. Fourier Transform Infrared Spectroscopy (FTIR) Analysis

**Figure 4:**
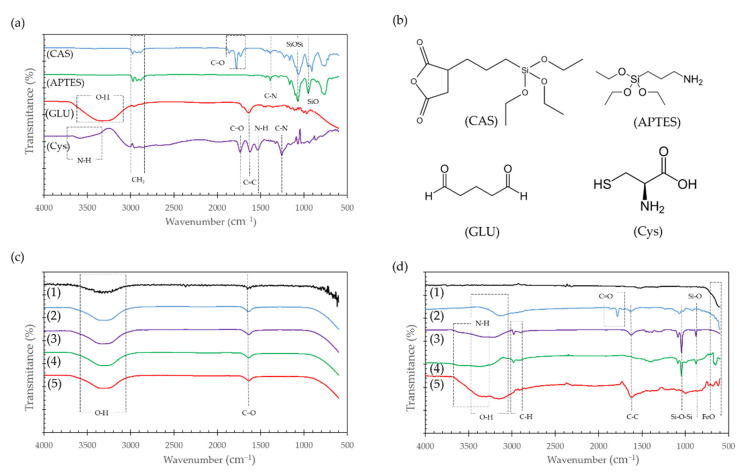
FTIR spectra (**a**) and chemical structure (**b**) of pure CAS, APTES, GLU and Cys. Additionally, FTIR spectra of (**c**) Al_2_O_3_ spheres and (**d**) Fe_3_O_4_ NPs. Each one functionalized with (2) CAS, (3) CASCys, (4) APTES and (5) APTES@GLU@Cys. The spectra of bare adsorbents were added for comparison (1).

FTIR analysis was performed to corroborate the effective surface modifications for the Al_2_O_3_ spheres and Fe_3_O_4_ NPs. Figure 4a,b show the IR spectra and chemical structure of the pure molecules used for the surface modification, while Figure 4c,d show the IR spectra of the substrates before and after their conjugation on the adsorbents.

The strong peaks near 1100 and 800 cm^−1^ found in pure APTES and CAS spectra (Figure 4a), are attributed to Si-O-Si and Si-O stretching vibrations, respectively [35,36]. These bands were also found in the modified Fe_3_O_4_ NPs as shown in Figure 4d and provided further evidence of the correct silanization of the surface. Peaks around 1800 cm^−1^ in pure CAS (Figure 4a) are due to C=O stretching of the anhydride group, that can also be seen on Fe_3_O_4_ NPs modified with CAS in Figure 4d. After the second modification with Cys to form the Fe_3_O_4_ NPs@CAS@Cys nanocompounds, these bands disappear when the anhydride group opens to covalently bind the Cys. In contrast, none of the modifications of Al_2_O_3_ spheres with the organo-silanes was confirmed by this method as the corresponding bands were absent in the spectra (Figure 4c).

A broad peak at around 3500–3000 cm^−1^ can be related to the O-H stretching vibration of the carboxyl group and the strong peaks close to 1620 cm^−1^ to the strong stretching vibration of C=C, which can also be found in the modified Fe_3_O_4_ NPs (Figure 4d). Of particular interest is the N-H stretching vibration present in APTES and Cys because it can be associated with the free amine groups of both molecules that have been thought to improve Cd adsorption. Figure 4d exhibited a broad absorption band at around 3200–3600 cm^−1^, which can also be attributed to the free amine groups after conjugation of both molecules on the Fe_3_O_4_ NPs. 

Bare Fe_3_O_4_ NPs in Figure 4d exhibited adsorption bands at around 700–600 cm^−1^, which can be attributed to the stretching vibration of the Fe-O bond of iron oxide [37]. The study of Al_2_O_3_ spheres with FTIR failed to show immobilization of any compound and only showed the C=O and O=H peaks related to surface oxidation, element traces or environmental H_2_O (Figure 4c). 

Weak bands at 3000–2700 cm^−1^ in the modified Fe_3_O_4_ NPs spectra (Figure 4d) were related to the C-H stretching vibration that can be also found in pure APTES, CAS and Cys (Figure 4a). In the particular case of Cys, this band could be also related to the stretching vibration of the S-H group [38].

### 2.2. Effect of Surface Functionalization on Cd Removal

**Figure 5:**
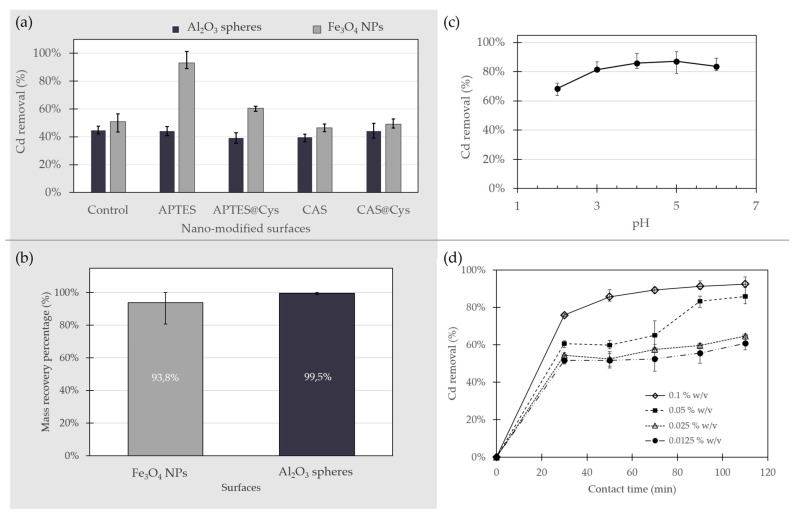
(**a**) Percentage of Cd removal in aqueous solution for Al_2_O_3_ spheres and Fe_3_O_4_ NPs. Removal efficiency (R%) was studied before and after surface modifications. (**b**) Adsorbent mass recovery after the removal assays. (**c**) Percentage of Cd removal at pH values in the range of 2.0–6.0 for Fe_3_O_4_ NPs @APTES. (**d**) Removal efficiency of Cd on Fe_3_O_4_ NPs @APTES as a function of contact time. Initial metal concentration of 0.1 mg/L; pH 5.0 and adsorbent dose in the range of 0.0125–0.1% (*w*/*v*).

The removal of Cd using the two inorganic surfaces with and without surface modifications was carried out in batch experiments at a 0.1 mg/L concentration of Cd in water. This experiment was intended to evaluate whether a particular surface modification showed a higher affinity for Cd in aqueous solution. The adsorption of the heavy metals is highly related to the properties of the adsorbent and the type of Cd ions species present in solution [18]. Figure 5a shows the removal efficiency of Cd by each adsorbent. The removal efficiencies of the control substrates without any surface modifications were below 51% in all cases. The best results for both functionalized and bare substrates were obtained for Fe_3_O_4_ NPs. The highest percentage of Cd removal of all the adsorbents was attained with Fe_3_O_4_ NPs functionalized with APTES, where Cd removal from aqueous solutions reached 94%.

Al_2_O_3_ spheres presented low removal efficiencies even after surface modification (Figure 5a). Bare Al_2_O_3_ spheres showed a Cd removal percentage of 44.7%, which was higher than any of the modified alumina spheres studied as evidenced by removal efficiencies as low as 39.4%. These results were expected, considering that the FTIR results showed no evidence of surface functionalization for Al_2_O_3_. Moreover, this substrate exhibited a highly irregular surface with low porosity and therefore lower superficial area for adsorption. As reported elsewhere, a nanoparticulated version of Al_2_O_3_ has already shown significant potential as an adsorbent for heavy metal removal from water [39,40], however, they are different in size, superficial area, and available active sites compared with the bulk counterpart used here. There are alternative possibilities for improving the functionalization by pre-activating the surface via hydrolyzation, spraying of the modifying agents, or by thermal treatments [41], however, these methodologies were not considered in this work.

There is marked interest from the scientific community in this magnetic nanoadsorbent for its unique properties, such as large surface area, low toxicity, high biocompatibility and simple separation and recovery from aqueous solutions with the aid of relatively low magnetic fields [18,36]. Unmodified Fe_3_O_4_ NPs showed a Cd removal efficiency of 50.8% (Figure 5a) similar to the 57.6% reported by Hosain et al. [29] for Fe_3_O_4_ NPs coated with nanochitosan but lower than the 96% reported by Chen et al. [30], using functionalized Fe_3_O_4_ nanoparticles with APTES. This suggests that their intrinsic ability for Cd removal could also be improved by increasing the affinity of the surface for the Cd ions. The already active surface of Fe_3_O_4_ NPs displays a significant number of hydroxyl groups, thereby it allows an effective functionalization with the organo-silanes to eventually improve the NPs’ capacity to bind Cd. In general, the Cd binding capacity of modified Fe_3_O_4_ NPs largely depends on the type of surface modification. The Fe_3_O_4_ NPs modified with APTES showed an improved Cd removal percentage, which is in contrast with CAS functionalization where a reduction was observed. The highest efficiency was 93.2% in ~18 h of exposure for Fe_3_O_4_ NPs @APTES, which is clearly superior to that achieved with other functionalized NPs. 

### 2.3. Substrate Recovery from Water

An ideal adsorbent should not only have a high adsorption capacity but induce reduced environmental impact. Here, we studied whether the adsorbents can be recovered from the treatment solutions after Cd removal to assess their potential reusability. We also wanted to evaluate if discharging back the recovered adsorbents to the environment might lead to possible environmental impacts. The Fe_3_O_4_ NPs were recovered by magnetic means while the Al_2_O_3_ spheres were filtered out from the solutions with a 500 µm pore size metallic filter. Figure 5b shows the mass percentage recovered after Cd removal tests for Fe_3_O_4_ NPs and Al_2_O_3_ spheres. Even though Al_2_O_3_ had the lowest capacity for Cd removal, they were effectively recovered from the solutions after their use. Al_2_O_3_ spheres showed the highest mass recovery of 99.5% in comparison with the 93.8% of the magnetic nanoparticles. However, considering the Cd removal results, Fe_3_O_4_ NPs @APTES was selected as the best material for Cd removal and was further used for additional tests and characterizations. The possible environmental effect of the approximately 6.2% of the non-recovered adsorbent was also evaluated.

### 2.4. Effect of Solution pH on Cd Removal

The removal of Cd using Fe_3_O_4_ NPs @APTES was studied for pH values in the range of 2.0–6.0 in experiments that lasted for 18 h. The type of ionized Cd species in solution is one of the most relevant parameters for effective adsorption. As reported by Jain et al. [23], Cd^2+^ is predominant in deionized water if the pH of the aqueous solution is lower than 7.0, and a subsequent increase in pH causes its precipitation as Cd(OH)_2_ [42].

The Cd removal efficiencies of the adsorbent at different pH values are shown in Figure 5c. Lower Cd removal percentages for Fe_3_O_4_ NPs @APTES were obtained at low pH values, which suggests that H^+^ ions in the aqueous solution are most likely bound to some of binding sites available on the material. Our results show a trend where cadmium removal increased as a function of pH. An increase in pH slightly improved Cd adsorption from 68.5% to 87.2% and reached a maximum at pH 5.0. For this reason, all the remaining experiments were carried out at this pH value. The decreased Cd capture above pH 6.0 could be related to precipitation Cd ions as Cd(OH)_2_.

### 2.5. Effect of Contact Time and Adsorbent Dose on Cd Removal

Adsorption time is important for understanding the adsorption mechanisms of the adsorbate. The effect of time on adsorption of Cd by Fe_3_O_4_ NPs @APTES is presented in Figure 5d. The percentage of Cd removal was evaluated for 110 min with four different doses of adsorbent, ranging from 0.0125% (*w*/*v*) to 0.1% (*w*/*v*). The removal of Cd increased rapidly in the first 30 min of exposure. The difference in concentration of Cd ions in the solution and the nanoparticles resulted in the fast diffusion of the element to the surface of the absorbent. This was followed by a regime of decreased adsorption for all the different studied doses of Fe_3_O_4_ NPs @APTES. For this adsorbent, a further increase in contact time had no significant effect on Cd removal after 90 min. The already occupied active sites in the adsorbent surface are charged positively by Cd and repel the free ions in the solution, thereby decreasing the adsorption rate. Cadmium removal at equilibrium increased as the adsorbent dose increased, most likely due to exposure to a higher superficial area with available binding sites for Cd ions [18,22]. The time evolution of adsorption was similar for almost all studied doses with the exception of 0.05% (*w*/*v*). Cd removal reached maxima in the range of 60.7% to 92.5% after 110 min, corresponding to removal ratios ranging from 0.49 to 0.09 mg of Cd per g of NPs, respectively.

### 2.6. Adsorption Isotherms

To investigate further the adsorption behavior of Fe_3_O_4_ NPs @APTES, we fitted the adsorption data to the widely used Langmuir and Freundlich models. 

The Langmuir model is frequently used to describe the deposition of a complete monolayer of the metal on the surface of the material via adsorption processes. This model assumes that the Cd ions are adsorbed by forming a homogeneous monolayer on the active sites in the surfaces of the adsorbent and there are no interactions between the adsorbed ions [32]. This is described by Equation (1) [43,44]:

(1) $$\frac{1}{q_{e}}=\left(\frac{1}{k_{L}q_{m}C_{e}}\right)+\frac{1}{q_{m}}$$

where $C_{e}\;\left({\mathrm{mg}\;g}^{-1}\right)$ is the concentration of adsorbate in the solution at equilibrium; $q_{e}\;\left({\mathrm{mg}\;g}^{-1}\right)$ is the adsorption capacity at equilibrium of the adsorbent surface; $k_{L}\;\left({L\;\mathrm{mg}}^{-1}\right)$ is the Langmuir isotherm constant and $q_{m}\;\left({\mathrm{mg}\;g}^{-1}\right)$ is the monolayer capacity of the adsorbent. According to this equation, the plot of $1/q_{e}$ vs. $1/C_{e}$ should be a straight line with slope $1/q_{m}$ and intercept$\;1/\left(k_{L}q_{m}\right)$. The Langmuir isotherm can be also expressed in terms of the dimensionless factor $R_{L}$, which is defined by Equation (2) [45]:

(2) $$R_{L}=\left(\frac{1}{1+k_{L}\times C_{o}}\right)$$

**Figure 6:**
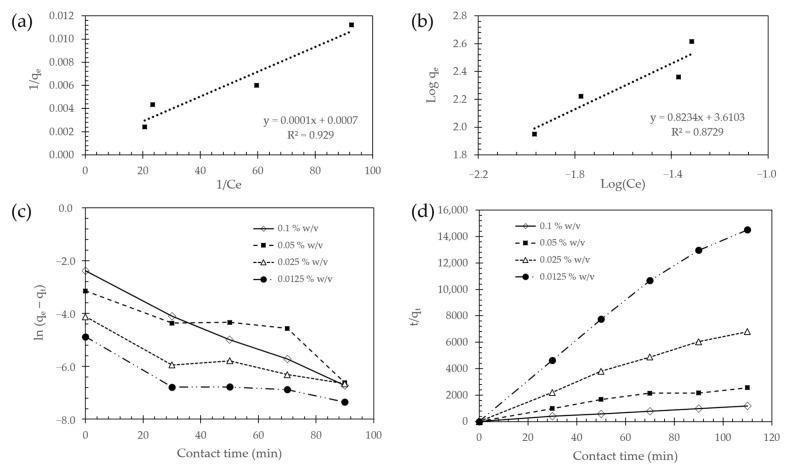
Linearized (**a**) Langmuir and (**b**) Freundlich adsorption isotherms of Fe_3_O_4_ NPs @APTES at 18 °C; (**c**) pseudo-first-order (PFO) and (**d**) pseudo-second-order (PSO) kinetic plots for Cd adsorption with Fe_3_O_4_ NPs @APTES.

The dominant adsorption mechanism can be deduced from the $R_{L}$ values, it can be either unfavorable$\;(R_{L}>1)$, linear$\;(R_{L}=1)$, favorable $\left(0<R_{L}<1\right)$ or irreversible$\;(R_{L}=0)$. Fe_3_O_4_ NPs adsorption yield a$\;R_{L}<1$, which indicates a favorable adsorption. The data fitted a linear adsorption process (Table 1) as evidenced with a correlation coefficient of 0.929 for Fe_3_O_4_ NPs @APTES. The Langmuir model fits relatively well the adsorption data for Fe_3_O_4_ NPs, which suggests that the assumption of a monolayer deposition might be correct for this adsorbent (Figure 6a). The $q_{m}$ value of Fe_3_O_4_ NPs @APTES is 1334.94 mg/g, which is reasonable compared with others reported previously by Javaheri, F. et al. [46] of 884.906 mg/g for Fe_3_O_4_@SiO_2_@m-SiO_2_–NH_2_ and 769.2 mg/g for nano zerovalent iron presented by Boparai et al. [47].

The Freundlich linear model is not restricted to a monolayer formation and includes the heterogeneous interaction between the material surface and the metal ion. This model indicates that the Cd ions are adsorbed on an uneven surface not only forming monolayers in the active sites but also uneven multilayers. Therefore, the energy distribution for the surface-active sites of the adsorbent is uneven in contrast with the Langmuir model [48]. The expression for Freundlich model is given by Equation (3) [35,45]:

(3) $$log\left(q_{e}\right)=log\left(k_{f}\right)+\frac{1}{n}\;\;log\left(C_{e}\right)$$

where $k_{f}\;\left(L\;g^{-1}\right)$ is the Freundlich constant and $n\;\left(g\;L^{-1}\right)$ is the constant exponent that describes the intensity of the adsorption process [35]. According to this equation, the plot of $log\;\left(q_{e}\right)$ vs. $log\;C_{e}$ should be a straight line with slope 1/n and intercept$\;K_{f}$.

The deviation from linearity for the Freundlich model can be estimated from n values. In this regard, this parameter can be either unfavorable (n<1), linear (n=1), favorable (n>1) or irreversible (n=0). Fe_3_O_4_ NPs yield a n>1, which could be associated with a favorable reaction [45]. The correlation coefficient was 0.873 for Fe_3_O_4_ NPs @APTES. Consequently, in comparison with the Langmuir model, the Freundlich one appears to describe the adsorption process less accurately for Fe_3_O_4_ NPs (Figure 6b).

Despite the fact that the Langmuir correlation coefficient ($R^{2}$= 0.929) is higher than the Freundlich model ($R^{2}$= 0.873), they are relatively low compared with similar reports in literature of 0.9973, 0.9950, 0.99 or 0.98 [32,48]. This is usually an indication that none of the constructed curves describes with high accuracy the adsorption behavior and there could be a more complex mechanism that governs the adsorption of Cd on this substrate. Other adsorption models such as the Dubinin−Radeshkevich may be able to provide a better mechanistic description of the process but the study of such a mechanism is beyond the scope of the present contribution.

### 2.7. Kinetic Studies

To describe the adsorption kinetics, the experimental data was fitted to both the pseudo-first-order (PFO) model and the pseudo-second-order (PSO) model. 

The PFO model is described by the following differential equation (Equation (4)) [49,50,51]:

(4) $$\frac{dq_{t}}{dt}=k_{1}\;\left(q_{e}-q_{t}\right)$$

where $q_{t}\;\left({\mathrm{mg}\;g}^{-1}\right)$ and $q_{e}\;\left({\mathrm{mg}\;g}^{-1}\right)$ are the metal ions adsorbed at time t (min) and at equilibrium, respectively, while $k_{1}\;\left(\min^{-1}\right)$ is the PFO rate constant. After integration between t=0 to t=t and q=0 to q=q the obtained expression is (Equation (5)):

(5) $$ln\left(q_{e}-q_{t}\right)=lnq_{e}-k_{1}t$$

The adsorption kinetics (Figure 6c and Table 2) were fitted to the PFO model by plotting $ln\left(q_{e}-q_{t}\right)\;$vs. t. The calculated parameters showed that the data failed to follow the PFO model for the studied surfaces and concentrations. The mean $R^{2}$ was 0.852 for Fe_3_O_4_ NPs @APTES, which indicates a substantial deviation from linearity. Such deviation becomes quite apparent at 10 min, which is most likely due to a fast initial uptake of metal ions [52].

The PSO model is described by the following differential equation (Equation (6)) [20,51]:

(6) $$\frac{dq_{t}}{dt}=k_{2}\;{\left(q_{e}-q_{t}\right)}^{2}$$

where $q_{t}\;\left({\mathrm{mg}\;g}^{-1}\right)$ and $q_{e}\;\left({\mathrm{mg}\;g}^{-1}\right)$ are the metal ions adsorbed at time t (min) and at equilibrium, respectively, while $k_{2}\;\left({g\;\mathrm{mg}}^{-1}{\;\min}^{-1}\right)$ is the PSO rate constant. After integration between t=0 to t=t and q=0 to q=q the obtained expression is (Equation (7)):

(7) $$\frac{t}{q_{t}}=\frac{1}{K_{2}q_{e}^{2}}-\frac{t}{q_{e}}$$

The parameters of the kinetic model were calculated by plotting $t/q_{t}$ vs. t. As shown in Table 3 and Figure 6d, the values of the calculated $q_{e}$ are similar to those found experimentally. The mean correlation coefficient ($R^{2}$) for Fe_3_O_4_ NPs @APTES was 0.976. This corroborated a much better fitting of the PSO model compared with the PFO one.

The concentrations used for this experiment were increased from 0.0125% (*w*/*v*) to 0.1% (*w*/*v*), which was the one selected to evaluate the other parameters, such as pH, reusability and surface functionalization. The lower concentration of adsorbent possibly explains why the PSO model showed a better data fit than the PFO [49]. Several reports have shown that by increasing the adsorbent’s initial concentration, the correlation of the experimental data with the PSO kinetics model decreases while it decreases for the PFO model [49]. The PSO model assumes that the rate of adsorption of metal ions on the surface is directly proportional to the available binding sites on the material. Consequently, for Fe_3_O_4_ NPs @APTEs, it is very likely that the adsorption process is highly dependent on the amount of Cd in direct contact with the surface of the substrates and the number of available active sites. In this work, the calculated and experimental values for $q_{e}$ in both PFO and PSO models are close to 0.007 mg/g, which is particularly small compared with those reported previously of 8.852 mg/g [48] and 9.13 mg/g [29]. This result could be explained by two of the initial conditions of the experiment. First, the low initial concentration of the stock Cd solution (0.1 mg/L), which is in some cases three orders of magnitude lower than those reported in other studies [29,30,48]. Second, the studied concentrations of adsorbent. Although our substrate had a removal efficiency of 94%, the maximum evaluated concentration was 0.1 mg/L and the ratio of initial free metal ions and available adsorption sites was relatively low for the studied doses of adsorbent. Subsequently, we suggest that we were most likely operating below the complete adsorption capacity of the material, and in therefore the values for the$\;q_{e}$ could change in higher initial cadmium concentrations.

### 2.8. Life Cycle and Desorption Studies

**Figure 7:**
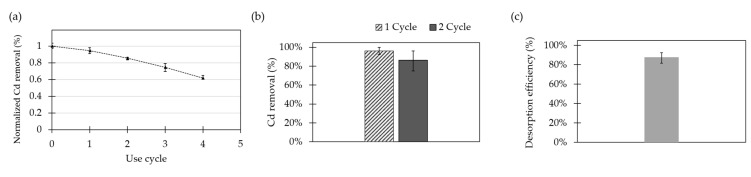
Percentage of Cd removal per cycle of Fe_3_O_4_ NPs @APTES (**a**) without desorption between cycles; (**b**) after desorption of surfaces by 0.1% (*v*/*v*) HCl solutions as regenerating agent; and (**c**) desorption efficiency after regeneration treatment.

The life cycle capacity of the adsorbent was estimated by conducting four cycles of Cd removal. The Cd removal efficiencies of the modified substrate per cycle without any desorption or recovery process in between cycles are shown in Figure 7a. The adsorbent showed a linear decreasing capacity for Cd adsorption after each cycle. The limit of reusability was established as an accumulated reduction in the adsorption capacity of 50%. For Fe_3_O_4_ NPs @APTES, the adsorption capacity was never reached for the conducted experiments; however, by following the linear tendency, it is expected to be close after the fifth cycle.

The ability to regenerate the surface is key for the economic and sustainable application of the developed adsorbents. For this reason, desorption studies were carried out with the aid of a 0.1% (*v*/*v*) HCl solution as the regenerating agent. The reusability of adsorbents was studied by evaluating the percentage of Cd removal before and after desorption (Figure 7b). Moreover, the desorption efficiency was calculated for Fe_3_O_4_ NPs @APTES (Figure 7c).

As reported by Katarina et al., in an acid medium such as HCl solution, Cd ions bound to chelating functional groups in the material are replaced by H^+^ [18]. For Fe_3_O_4_ NPs @APTES, the attained desorption efficiency was 87.6 ± 5.4%. Furthermore, on the second use cycle, the Cd adsorption percentage for Fe_3_O_4_ NPs @APTES decreased from 96.1 ± 3.9% to 86.4 ± 6.8%. In general, this indicates that this nanocompound shows good reusability for cyclical operation at the reported concentrations.

### 2.9. Environmental Repercussion

#### 2.9.1. Phytotoxicity

**Figure 8:**
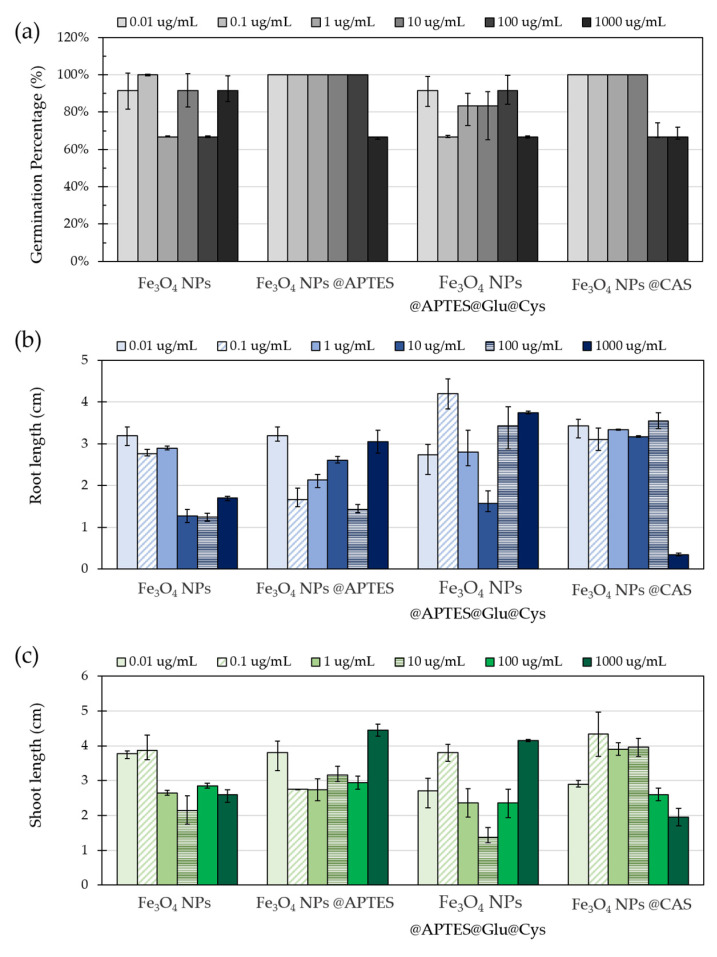
(**a**) Germination percentage, (**b**) root and (**c**) shoot length of ryegrass seed after 6 days of exposure to bare Fe_3_O_4_ NPs and Fe_3_O_4_ NPs functionalized with APTES, APTES@Glu@Cys and CAS.

A simple, rapid, sensitive, and low-cost method to evaluate NP toxicity is through the study of seed germination and root elongation of plant seeds [53]. Figure 8 shows the impact on seed germination, root and shoot growth of NPs at six different concentrations in the range of 0.1 to 1000 µg/mL. Seed germination was recorded after the fourth and seventh day of exposure. The results were then used to calculate the percentage of germination for each treatment. Seed germinations appeared largely unaffected by the NPs as evidenced by values above 60% for all evaluated concentrations (Figure 8a). Although the multiple comparison tests seemed to show no significant differences between the evaluated groups (with *p* = 0.198), Levene’s test showed *p* = 0.007, thereby suggesting that a least one of the treatments was different, which was corroborated by applying a Tukey test where the Fe_3_O_4_ NPs @APTES@GLU@Cys treatment was found to be statistically different from the others. The treatment that seemed to affect germination the most was Fe_3_O_4_ NPs @APTES@GLU@Cys with a value 6.6% below the average (i.e., about 87%). This was followed by Fe_3_O_4_ NPs with a value 2.4% below the average. In contrast, the remaining treatments showed germination values above the average. Fe_3_O_4_ NPs @CAS was indeed 1.7% above the average, and finally, Fe_3_O_4_ NPs @APTES was 7.35% above the average. Concentrations between 0.01 and 10 µg/mL showed no significant differences in germination, but those at 100 µg/mL and above led to germination changes in a concentration dependent manner.

The root and shoot elongation (Figure 8b,c) were affected differently by each adsorbent. Changes in root elongation were observed in the roots of each seed. Multiple comparisons between treatments for the longest root showed a *p* = 0.04, indicating that at least one of them was statistically different. Here, the group of seeds treated with unmodified Fe_3_O_4_ NPs showed the lowest elongations in its main root with up to 0.39 cm below the average length (i.e., 2.43 cm). Even though for NPs modified with APTES, the root and shoot elongations were not significantly inhibited, the effect of the treatment was still unclear. A slight inhibition of shoot and root growth was observed for Fe_3_O_4_ NPs @CAS at 10 µg/mL but surprisingly, not at the highest evaluated concentration.

Overall, Fe_3_O_4_ NPs showed no observable phytotoxicity except for the highest concentration, i.e., 1000 µg/mL. If NPs are to be used for wastewater treatment at a concentration of 0.1% (*w*/*v*), the approximate concentration of lost NPs in the environment, according to the reported WR%, would be approximately 62 µg/mL. At this concentration, no evident phytotoxicity effects on germination, root length, or shoot elongation were observable, which suggests a potentially low environmental impact. However, large-scale experiments with long-term exposure to realistic concentrations of Fe_3_O_4_ NPs are still needed to come to a definitive conclusion [54].

#### 2.9.2. NPs Toxicity in Zebrafish Embryos

**Figure 9:**
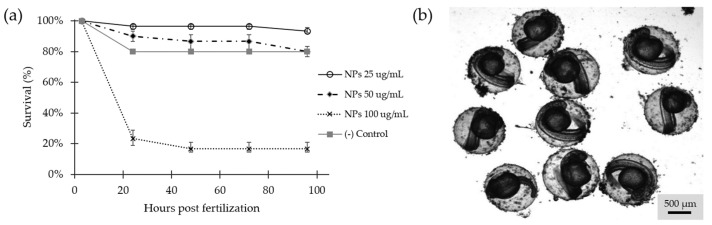
(**a**) Survival rate for embryos exposed to different concentrations of Fe_3_O_4_ NPs @APTES (25, 50 and 100 µg/mL) compared with the control group. Data are showed as percentage of survival (*n* = 30 embryos for each concentration and a control that was normalized to the highest survival well). (**b**) Stereoscope image of 48 hpf embryos exposed to Fe_3_O_4_ NPs @APTES at 25 µg/mL.

Survival in zebrafish embryos was determined by observation of activity and movement, heart rate, and changes in blood circulation [55]. Survival was evaluated from 3 to 96 hpf (Figure 9a) showing statistically significant differences (*p* = 3 × 10^−12^). Even though the effect of the exposure to the NC was concentration-dependent, the concentration of 25 and 50 µg/mL, showed minimal mortality rates and no evidence of malformations, while exposure to 100 µg/mL was highly lethal. According to the OECD guide, this result suggests that concentrations of 25 and 50 µg/mL present in the water, should not be considered harmful [56]. During the treatment, accumulation of NPs was observed in the chorion, which became more evident as the concentration increased (Figure 9b). However, this experiment was insufficient to determine if the observed accumulation led to significant changes in the normal development of the embryos or if hatching was inhibited by blockage of the chorionase activity [57], the presence of acids in the medium [58], and/or changes in hypoxia [59].

## 3. Materials and Methods

### 3.1. Materials

Iron (III) chloride hexahydrate (97%) (FeCl_3_∙6H_2_O), tetramethylammonium hydroxide (TMAH) (25%), (3-Aminopropyl) triethoxysilane (APTES) (98%) and L-Cysteine (Cys) (97%) were purchased from Sigma-Aldrich (St. Louis, MO, USA). Iron (II) chloride tetrahydrate (98%) (FeCl_2_∙4H_2_O), glutaraldehyde (25%) (GLU), Cd standard solution 1000 mg/L and sodium hydroxide (NaOH) (98%) were obtained from PanReac AppliChem (Barcelona, Spain). Additionally, 3-(triethoxysilyl) propylsuccinic anhydride (CAS) (95%) was purchased from Shanghai Kayi Chemical Co. (Shanghai, China). Commercial aluminum oxide spheres (Alumina) between 600–800 µm of diameter were acquired from Torrecid (L’Alcora, Castellón, Spain).

### 3.2. Manufacture and Functionalization of Surfaces

#### 3.2.1. Synthesis of Fe_3_O_4_ NPs

Nanoparticles were synthetized by co-precipitation method, using 2 M FeCl_3_∙6H_2_O and 1M FeCl_2_∙4H_2_O, diluted in milli-Q water. Solutions were then magnetically stirred and subsequently dripped to a 10 mL solution 8 M of NaOH with a syringe pump 78-8110C Programmable Touch Screen (Cole-Parmer, Vernon Hills, IL, USA) at 0.2 mL/min of infusing flow rate for 60 min. At the same time, the solution was stirred at 1500 rpm and 90 °C in the stirring hot plate. The final solution was left to precipitate with the aid of a neodymium magnet for 12 h. The resulting supernatant was discarded, and the precipitate was re-dispersed and washed adding milli-Q water, until pH 7.0 was reached.

#### 3.2.2. Surface Functionalization of Substrates

Substrates were functionalized by silanization with two different organo-silane molecules, APTES and CAS. Surface hydrolysis was used to promote silane immobilization by exposing overnight the substrates to a pH 11.0 solution. The solution was prepared using milli-Q water and adding drops of 0.5 M NaOH until reaching pH 11.0. For Fe_3_O_4_ NPs silanization, 10mL of 0.5% (*v*/*v*) APTES or CAS solution was added to 20 mL aqueous suspension of Fe_3_O_4_ NPs 0.016 g/mL and sonicated for 10 min. Similarly, 2 g of activated Al_2_O_3_ spheres were dispersed in solutions of APTES or CAS 0.2% (*v*/*v*). All the reactions were carried out for 24 h at room temperature. Next, the functionalized surfaces were washed several times with abundant milli-Q to remove excess reagents. 

**Figure 10:**
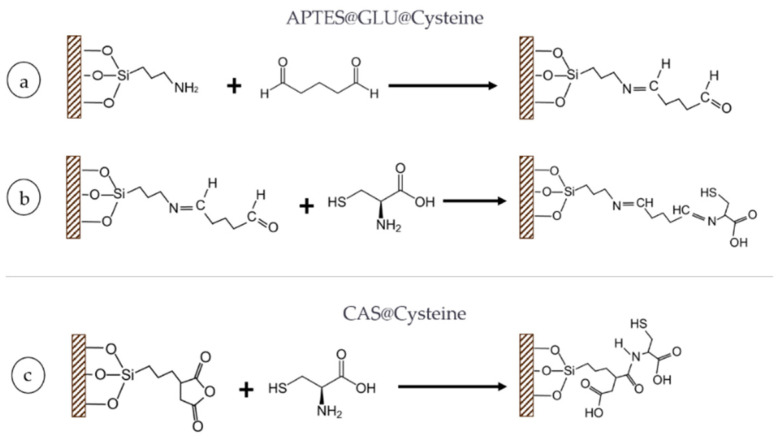
Schematic representation of the surface modifications with l-Cysteine. Substrate modification with (**a**) first step of APTES followed by Glutaraldehyde, (**b**) second step of APTES@GLU and then L-Cysteine, and (**c**) CAS and then L-Cysteine.

Additionally, a second functionalization with l-Cysteine was carried out by following two different functionalization schemes (Figure 10). Previously silanized substrates (with APTES), were first allowed to activate in a solution of GLU 1% (*v*/*v*) for 30 min. This was followed by thoroughly rinsing the substrate with milli-Q water to avoid nonspecific adsorption of the GLU. Alternatively, substrates silanized with CAS followed a succinylation of the anhydride group with the terminal amine group of Cys. The reaction was conducted under a basic pH conditions (i.e., pH: 8.0–10.0). Both CAS and APTES@GLU substrates were subsequently reacted with Cys at 0.01% (*w*/*v*) overnight (Figure 10b,c). All reactions were carried out at room temperature.

### 3.3. Adsorbent Characterization

The hydrodynamic diameter of the synthesized Fe_3_O_4_ NPs was determined at room temperature by Dynamic Light Scattering (DLS) using a Zetasizer Nano ZS, (Malvern Panalytical, Malvern, Worcestershire, UK). This was accomplished by resuspending the magnetic NPs at 3% (*w*/*v*) in 1 mL of milli-Q water in the presence of 100 µL TMAH. Morphology and composition of both substrates before and after functionalization were studied with focused ion beam-scanning electron microscope (FIB-SEM). For the measurement, the samples were placed on aluminum-coated slices previously covered with carbon tape and then coated by evaporation with a thin film of Au to improve resolution and signal to noise ratio. Imaging was carried out under vacuum at a beam potency of 10 kV and 5000X of magnification with a Jeol Lyra 3 (TESCAN, Brno, Czech Republic). The surface modification of Fe_3_O_4_ NPs, and Al_2_O_3_ spheres with APTES, CAS, Glutaraldehyde and L-Cysteine were also verified via Fourier transform infrared spectroscopy (FTIR) using a Bruker Alpha II FTIR Eco-ATR (Bruker, Ettlinglen, Germany). Spectra were collected in the range of 4000–500 cm^−1^ with a spectral resolution of 2 cm^−1^ at room temperature.

### 3.4. Cd Removal Studies

Adsorption experiments were carried out in batches by maintaining 10 mg of substrate per 10 mL of Cd solution (0.1% (*w*/*v*) overnight (~18 h) at room temperature) and 200 rpm agitation in a VWR Standard orbital shaker (Avantor, Radnor, PA, USA). Control samples of the original Cd solution were taken prior to Cd removal assays and all measurements were conducted in triplicate. After the removal experiments, Fe_3_O_4_ NPs were recovered using a strong neodymium magnet. Al_2_O_3_ spheres were separated and recovered from the solutions with a 500 µm pore size metallic filter.

A stock solution of 0.1 mg/L of Cd was prepared from a 1000 mg/L Cd standard solution with milli-Q water. The Cd concentration in the solutions was measured via flame atomic adsorption spectroscopy (FAAS). Cadmium removal efficiency (R%) for each substrate was calculated according to the following equation (Equation (8)):

(8) $$R\%=\frac{C_{o}-C_{f}}{C_{o}}\times 100$$

where $C_{o}$ is the initial concentration of the Cd ion in the stock solution (mg/L); and $C_{f}$ is final concentration of the Cd ion in the treated solution (mg/L).

The amount of Cd ions removed on the substrates was calculated as (Equation (9)):

(9) $$q=\frac{C_{o}-C_{f}}{w}\times V$$

where *q* is the adsorption capacity (mg/g); *w* is the substrate dosage (g); and *V* is the total Cd solution volume (L).

#### Substrate Recovery

All the modified surfaces were weighted before and after the removal experiments. The percentage of mass recovered of each adsorbent after the removal experiments was calculated by the following relationship (Equation (10)):

(10) $$WR\;\%=\frac{W_{f}}{W_{o}}\times 100$$

where *WR*% is the percentage of mass recovered; $W_{o}$ is the initial substrate dosage; and $W_{f}$ is the mass of filtered substrate from solutions after the removal experiments.

### 3.5. Impact of Substrate Type and Surface Modifications

The impact of the type of substrate and surface modification was investigated via adsorption experiments of Cd solution at a 0.1 mg/L concentration. The pH of the solutions was adjusted to 5.0 and the temperature maintained at room level (18 °C). The concentration of the remaining Cd ions was measured by FAAS after the substrates were filtered out with a 0.45 µm PTFE filter.

### 3.6. Effect of Solution pH

The adsorbents exhibiting the highest removal efficiencies (R%) were selected to investigate further the impact of pH on adsorption. In this regard, adsorption experiments were conducted at pH values ranging from 2.0 to 6.0 adjusted by adding either 0.1 M HCl or 0.1 M NaOH solutions. The initial Cd concentration for these studies was 0.1 mg/L.

### 3.7. Kinetic Studies

In these experiments, silanized Fe_3_O_4_ NPs (Fe_3_O_4_ NPs@APTES) at four different concentrations ranging from 0.0125 to 0.1% (*w*/*v*) were employed for removal experiments as described above. After the experiments, the adsorbents were recovered as explained above and the collected supernatants measured for cadmium contents as a function of time for up to 2 h.

### 3.8. Reusability Studies

The reusability of the Fe_3_O_4_ NPs@APTES was analyzed by carrying out four full operation cycles of 0.1 mg/L Cd removal at pH 4.0. After each cycle, the material was removed from the solution, transferred to a fresh Cd solution, and maintained therein overnight. Cd removal was measured with respect to the control solution before and after exposure for a period of 5 days.

Desorption studies were performed to investigate the feasibility for regeneration and reusability of the substrates. The Cd removal assays for Fe_3_O_4_ NPs@APTES started at pH 5.0 and at a Cd concentration of 0.1 mg/L. Then, adsorbents were separated, recovered, and placed independently for 1 h in a 0.1% (*v*/*v*) HCl solution followed by rinsing multiple times with milli-Q water to desorb Cd ions from their surfaces. The regenerated adsorbents were added later on to fresh Cd solutions under the same conditions to compare their removal efficiencies with those of pristine materials. The desorption efficiency was calculated using the following equation (Equation (11)) [18]:

(11) $$Desorption\;efficiency\;\left(\%\right)=\frac{C_{de}}{C_{a}}\times 100$$

where $C_{a}$ (mg/L) is the concentration of the originally adsorbed Cd ions, and $C_{de}$ (mg/L) is the concentration of Cd in the solution after the desorption process.

### 3.9. Potential Environmental Impact

Different toxicity tests were conducted for the adsorbents with the highest Cd removal efficiencies to assess their potential environmental impact before their discharge to superficial waters.

#### 3.9.1. NPs Phytotoxicity

Fe_3_O_4_ NPs phytotoxicity was determined by measuring the germination and stem length of *Lolium perenne* seeds (Ryegrass) (Agrosemillas, Medellin, Colombia) placed on a filter paper in culture plates. Solutions with unmodified NPs and modified with APTES, APTES@Cys and CAS at six different concentrations (0.01, 0.1, 1, 10, 100, 1000 µg/mL) in water were added to the culture plates with 6 seeds per well. Seeds were grown for seven days under static conditions at 18 °C and light cycles of 10 h.

#### 3.9.2. NPs Toxicity in Zebrafish Embryos

Adult, TAB wild type, zebrafish were maintained under a constant 14 h light–10 h dark cycle at 28 °C, according to standard protocols [60] in a controlled multitank recirculating system (Aquaneering, San Diego, CA, USA). The day before eggs were required for the assay, adults were placed in breeding tanks and allowed to breed. Embryos were collected 1 h after light was turned on the next morning and raised on 6-well microplates in egg water (60 µg/mL sea salt in RO water with 1 mg/L Methylene Blue) at 28 °C until 3 h post fertilization (dpf).

Groups of 10 embryos (3hpf) were exposed to Fe_3_O_4_ NPs @APTES at different concentrations (25, 50, 100 mg/L) until 4 dpf. Egg water was used as negative control. Each treatment was performed in triplicate, under the same conditions, and every 24 h, the water with the treatment was changed according to the protocols indicated by the OECD guide [56].

Survival of the embryos was determined by observations of movement, heartbeat, and blood circulation aided by a stereoscope microscope AZ100M (Nikon, Tokyo, Japan) at 3, 24, 48, 72, and 96 h. Embryos that appeared opaque and exhibited white color were considered coagulated and were removed from the tank. All protocols were approved by the Institutional Animal Care and Use Committee of Universidad de los Andes (CICUAL) with the C.FUA_19-004.

#### 3.9.3. Statistical Analysis

Germination of seeds, root and shoot elongation data were analyzed via a two-way ANOVA (with NP type and concentration as factors) followed by a Bonferroni post hoc test and were expressed as the mean ± standard deviation (S.D.).

Survival rate was determined during acute toxicity test (4 days) and was tested for Fe_3_O_4_ NPs @APTES, together with negative control (egg water). The collected data were analyzed with one-way ANOVA followed by Tukey test and were expressed as the mean ± S.D. The statistical analyses were developed in Minitab 19 Statistical Software, and for all comparisons, the level of significance was established as *p* ≤ 0.05.

## 4. Conclusions

Due to the increasing amounts of cadmium and other heavy metals being released into the environment on a daily basis, more efficient and inexpensive routes for their capture are urgently needed. One interesting strategy to address this need is to develop potent adsorbents capable of capturing significant amounts of heavy metal ions, and with the potential of being reused several times. Here, we explored the use of bare and functionalized Fe_3_O_4_ NPs and Al_2_O_3_ spheres as potential cadmium adsorbents. The already remarkable capacity of Fe_3_O_4_ for Cd adsorption was improved in more than 20% by surface functionalization with the organo-silane APTES. This was attributed to the significant increase in surface reactivity given by the pendant amine groups of APTES. For Fe_3_O_4_ NPs, an optimum pH of 5.0 was found for Cd removal, which was attributed to suitable charges for strong electrostatic interactions between Cd ions in the solution and the surface functional groups. Kinetic studies of cadmium adsorption suggest that the process is most likely controlled by the available active sites on the surface of the materials as well as by the cadmium ion concentrations in solution. All adsorbents can be easily recovered from water solutions with efficiencies higher than 80%; however, despite having a relatively lower cadmium capturing capacity, Al_2_O_3_ spheres showed the best results. Fe_3_O_4_ NPs @APTES recycled up to four uses with more than 50% of their removal efficiency. No evident phytotoxicity was found for either the bare or functionalized adsorbents for concentrations below 100 µg/mL. Fe_3_O_4_ NPs @APTES evaluated here showed that toxicity is concentration dependent. Survival data suggests exposures to concentrations below 50 µg/m are not harmful; however, more detailed studies that includes the effect on hatching, malformation, changes in behavior and possible nanoparticle bioaccumulation, among others; are suggested as future work.

Overall, these results indicate that metal ion retention is highly dependent on the porosity of the surface and the adsorbent´s particle size. Similar cadmium removal percentages were achieved with both Fe_3_O_4_ and Al_2_O_3_. Further studies at a larger scale are required to evaluate whether the adsorbents can be potentially introduced into niche markets locally and internationally.

## Figures and Tables

**Table 1:** Langmuir and Freundlich models parameters recovered for Fe_3_O_4_ NPs @APTES.

Langmuir Model		Freundlich Model	
Isotherm Parameters	Values	Isotherm Parameters	Values
$q_{m}$	1334.96	n	1.214
$K_{L}$	7.002	$K_{f}$	36.979
$R_{L}$	0.588	-	-
$R^{2}$	0.929	$R^{2}$	0.873

**Table 2:** Experimental and PFO parameters for Fe_3_O_4_ NPs @APTES at four different substrate concentrations % (*w*/*v*). [$C_{o}\;$= 0.1 mg/L; mixing rate: 200 rpm; T = 18 °C; pH = 5.0].

Parameters	Fe_3_O_4_ NPs @APTEs Concentrations % (*w*/*v*)			
	0.0125%	0.025%	0.05%	0.1%
$q_{e}\;\mathrm{calculated}$	0.0046	0.0107	0.0469	0.0806
$K_{1}\;\mathrm{calculated}$	0.0240	0.0256	0.0323	0.0472
$q_{e}\;\mathrm{experimental}$	0.0076	0.0162	0.0429	0.0925
$R^{2}$	0.7719	0.8370	0.8043	0.9936

**Table 3:** Experimental and PSO parameters for Fe_3_O_4_ NPs @APTES at four different substrate concentrations % (*w*/*v*). [$C_{o}\;$= 0.1 mg/L; mixing rate: 200 rpm; T = 18 °C; pH = 5.0].

Parameters	Fe_3_O_4_ NPs @APTEs Concentrations % (*w*/*v*)			
	0.0125%	0.025%	0.05%	0.1%
$q_{e}\;\mathrm{calculated}$	0.0074	0.0160	0.0439	0.0942
$K_{2}\;\mathrm{calculated}$	31.8227	12.5918	1.9662	2.9968
$q_{e}\;\mathrm{experimental}$	0.0076	0.0162	0.0429	0.0925
$R^{2}$	0.9877	0.9865	0.9336	0.9962

## Data Availability

The data and contributions presented in the study are included in the article. Further inquiries can be directed to the corresponding author.

## Sample Availability

Samples of the compounds are not available from the authors.
